# Current Hypothesis for the Relationship between Dietary Rice Bran Intake, the Intestinal Microbiota and Colorectal Cancer Prevention

**DOI:** 10.3390/nu8090569

**Published:** 2016-09-15

**Authors:** Winnie K. W. So, Bernard M. H. Law, Patrick T. W. Law, Carmen W. H. Chan, Sek Ying Chair

**Affiliations:** The Nethersole School of Nursing, The Chinese University of Hong Kong, Shatin, the New Territories, Hong Kong, China; winnieso@cuhk.edu.hk (W.K.W.S.); patricklaw@cuhk.edu.hk (P.T.W.L.); whchan@cuhk.edu.hk (C.W.H.C.); sychair@cuhk.edu.hk (S.Y.C.)

**Keywords:** colorectal cancer, rice bran, intestinal microbiota, microbial dysbiosis

## Abstract

Globally, colorectal cancer (CRC) is the third most common form of cancer. The development of effective chemopreventive strategies to reduce CRC incidence is therefore of paramount importance. Over the past decade, research has indicated the potential of rice bran, a byproduct of rice milling, in CRC chemoprevention. This was recently suggested to be partly attributable to modification in the composition of intestinal microbiota when rice bran was ingested. Indeed, previous studies have reported changes in the population size of certain bacterial species, or microbial dysbiosis, in the intestines of CRC patients and animal models. Rice bran intake was shown to reverse such changes through the manipulation of the population of health-promoting bacteria in the intestine. The present review first provides an overview of evidence on the link between microbial dysbiosis and CRC carcinogenesis and describes the molecular events associated with that link. Thereafter, there is a summary of current data on the effect of rice bran intake on the composition of intestinal microbiota in human and animal models. The article also highlights the need for further studies on the inter-relationship between rice bran intake, the composition of intestinal microbiota and CRC prevention.

## 1. Introduction

Despite our increased knowledge of the causes of cancer from a molecular perspective, cancer has still been responsible for a significant number of deaths worldwide in recent years. Among all forms of the disease, colorectal cancer (CRC) is ranked third as the most common worldwide. More than 1.36 million new CRC cases were reported in 2012, with a mortality rate of nearly 700,000 [1]. Further, CRC risks were thought to be associated with age. The likelihood of CRC development was reported to increase dramatically by the age of 50 [2]. With the aging of the world’s population, the global burden of CRC is therefore expected to increase in the coming years. In order to reduce this burden, the development of effective preventive strategies against CRC is of paramount importance.

Although the risk factors for CRC are still not fully understood, there has been evidence supporting the role of lifestyle behaviour in the modification of CRC risks. Among the various forms of lifestyle behaviour, diet has received a considerable level of attention. For example, high consumption of processed meat has been shown to be linked to increased CRC risk [3,4,5]. On the other hand, frequent consumption of fruits reduces the risk of developing colorectal carcinoma [6]. Dietary fibre intake was also suggested to be linked to a lower risk of CRC, although there is some controversy over these observations [7,8]. All these data indicate that CRC prevention could be achieved through the intake of certain dietary supplements containing nutrients that may lead to a lowered CRC risk. Among these dietary supplements, rice bran is one of the most promising potential candidates for use in cancer chemoprevention.

Rice bran is one of the byproducts of the milling process of rice, a staple food that is consumed widely throughout the world, especially in Asia and Latin America. The majority of rice is grown in Asia, accounting for about 90% of the world’s total rice production [9]. With the abundant production of rice in Asia, rice bran is readily available at low cost in Asian countries. Moreover, its high nutritional value has strengthened its potential as a food supplement to improve health. In recent years, there has been a dramatic increase in the number of studies supporting the potential of this low-cost, health-promoting dietary supplement in the chemoprevention of various cancers, especially CRC. For example, rice bran has been found to contain a variety of phytochemicals and nutrients known to exhibit anti-tumour properties through their effects on various cellular pathways, such as the inhibition of oxidative damage, control of cell cycle progression and apoptotic induction. Studies of how the phytochemicals and nutrients in rice bran confer their beneficial effects in cancer prevention, including the molecular mechanisms involved, were previously reviewed by Henderson and colleagues [10]. 

More recently, several studies have also shown that rice bran intake modifies the intestinal microbiota in a way that promotes intestinal health, and potentially chemoprevention of CRC. The mechanisms by which this is achieved, and how such modification may lead to lower risks of CRC development, has therefore become a burgeoning field of research in recent years, and further inquiry into the topic is clearly warranted. 

The present review attempts to summarise the results of previous research that show how the modification of intestinal microbiota is linked to CRC, and those that report the effect of rice bran intake on the composition of intestinal microbiota, in order to establish the current evidence for the hypothesis that rice bran intake may promote CRC chemoprevention by modifying the composition of intestinal microbiota. The review will also point to the current knowledge gap concerning the relationship between the intake of rice bran, the modification of intestinal microbiota, and CRC chemoprevention, in order to stimulate further research on the topic. 

## 2. Evidence for the Link between Intestinal Microbiota and CRC

In the past decade, much research had been carried out investigating the relationship between intestinal microbiota and cancer. Indeed, it has been suggested that bacteria residing in the human gut, which amount to trillions with over 1000 reported bacterial species [11], do affect human health [12], with such effects thought to be at least partly attributable to the metabolic activity of certain species of intestinal bacteria. For example, fermentation of plant-based carbohydrates by intestinal bacteria leads to the generation of short-chain fatty acids (SCFAs) such as butyrate. This compound was thought to be the energy source for the colonic mucosa [13], and to play a role in the inhibition of inflammation and tumourigenesis [14]. Butyrate has been shown to upregulate the expression and activation of glutathione-*S*-transferase [15], an enzyme involved in the prevention of oxidative stress. Further, butyrate has been revealed as a histone deacetylase inhibitor, which acts to promote histone acetylation and modulate the expression of a variety of genes involved in inflammation, cell cycle progression, and apoptosis, processes that play roles in carcinogenesis [14,16]. This gives butyrate its anti-tumour properties. Its increased production in the intestine is therefore thought to promote intestinal health and a lowered risk of CRC. The mechanisms of how SCFAs contribute to intestinal health have recently been reviewed by Zeng and colleagues [17].

Since SCFAs are produced by intestinal bacteria, it is generally considered that the composition of intestinal microbiota, such as the abundance of butyrate-producing bacteria in the intestine, can affect intestinal health and the risks of CRC. Indeed, we currently have a wealth of data that supports the association between changes in the composition of intestinal microbiota, or microbial dysbiosis, and CRC risk. Here, we provide an overview of the studies that demonstrate this association, and the current evidence on how microbial dysbiosis may contribute to CRC development. 

### 2.1. Composition of Intestinal Microbiota Is Implicated in CRC Risks

Indeed, previous research has suggested that changes in the intestinal abundance of certain bacterial genera may lead to modifications in CRC risks. To lend support to this hypothesis, butyrate-producing bacteria have been shown to represent a significant proportion of bacterial species in a healthy gut. However, a marked decrease in the population of such bacteria was observed in CRC patients [18,19,20]. In addition to this, some probiotic species, such as certain bacterial genera belonging to the class of Clostridium (*Faecalibacterium*), have been shown to be fewer in number among CRC patients [21,22]. Moreover, health-promoting *Bifidobacteria* were also found to be under-represented both in CRC patients [22] and in a CRC mouse model [23]. These findings indicate that CRC is associated with a lower intestinal abundance of health-promoting bacteria. 

In contrast, the population of a number of pathogenic bacterial phyla was found to have increased in the gut of CRC patients and animal models. For example, *Fusobacteria* were revealed to be present in higher abundance in the intestines [24,25], tumours [26], and stool samples [27,28] of CRC patients and in a CRC mouse model [23]. Rubinstein and colleagues [27] went further and revealed that *Fusobacteria* might directly contribute to carcinogenesis through the expression of an adhesin named FadA, which can stimulate cell proliferation through a cellular signalling mechanism involving E-cadherin and β-catenin. Other pathogenic species, such as *Bacteroides fragilis* [19,29], *Enterococcus faecalis* [30], and *Streptococcus gallolyticus* [31], were also shown to be more enriched in the stool samples of CRC patients. Likewise, the population of some enteropathogenic bacteria, such as those from the *Escherichia* and *Shigella* genera and the *Enterococcaceae* family, was shown to be larger in the intestines of CRC patients [19,23,32,33]. *Akkermansia muciniphila*, a bacterial species found to degrade mucin that plays a role in the protection of the intestinal mucosal layer [34], was also found to show a four-fold increase in number in the stool samples of CRC patients [20]. Such data therefore support the involvement of microbial dysbiosis in CRC and a possible involvement of the above pathogenic bacteria in CRC pathogenesis.

Further support for the contribution of microbial dysbiosis to CRC development may also be demonstrated by the alpha bug model and the bacterial driver–passenger model, described by Sears and Pardoll [35] and Tjalsma et al. [36] respectively. In the alpha bug model, certain bacterial species, such as enterotoxigenic *Bacteroides fragilis*, may induce changes in the intestinal mucosa that favour tumour progression, including epithelial cell proliferation and induction of inflammation. Meanwhile, these bacteria can take the lead in modifying the colonic bacterial community so that the proportion of health-promoting bacteria will be dramatically decreased. All these events thereby lead to a favourable environment for tumour progression [35]. In the bacterial driver–passenger model, it is hypothesised that pathogenic bacteria would start colonising the gut in individuals prone to developing CRC, resulting in a significant decrease in the proportion of health-promoting bacteria such as *Bifidobacteria* and butyrate-producing bacteria in the intestine. These pathogenic bacteria, known as bacterial drivers, will elicit an inflammatory process in the gut, causing intestinal inflammation, gene mutations, and hyperproliferation of colonic cells, which lead to the formation of an adenoma. Such events will further cause an increase in the epithelial permeability of the intestinal wall, modifying the microenvironment of the microbiota where the adenomas form. These changes may lead to a replacement of the bacterial drivers by certain opportunistic pathogens, or bacterial passengers, to colonise in the intestine, thereby exacerbating the tumourigenesis process [36]. In other words, a temporal change in the composition of intestinal microbiota is involved in CRC progression, and such changes appear to contribute to the formation of tumours in the intestine.

However, there is some controversy over the changes in the number of certain bacterial genera in CRC patients and animal models. For instance, bacteria belonging to the group *Bacteroides-Prevotella* were found in a study to be present in higher abundance in the stool of CRC patients than in those of normal individuals [29]. However, in other studies the number of *Prevotella* present in the intestine of a rat model of CRC was shown to be 30% smaller than that in healthy rats [37,38]. A further study in China revealed that mucosal tissue samples obtained from CRC patients contained a lower number of *Proteobacteria* [25], a pathogenic bacterial phylum. On the other hand, an American study showed that *Proteobacteria* were present in higher abundance in the colorectal biopsy samples of CRC patients than in those of controls [39]. While we cannot rule out differences in the methodologies used in these studies, discrepancies could be explained by the ethnicity of the human subjects involved in the studies, and possible differences in the properties of different bacterial species and strains in the same bacterial genera. Further work is therefore recommended to bring forward more evidence of the changes in the abundance of various bacterial species during CRC development, with a focus on the comparison of the relative abundance of health-promoting and pathogenic bacteria in CRC patients and healthy individuals. However, it is clear that microbial dysbiosis is a contributory factor to CRC pathogenesis. This is further supported by the recent discovery that frequent use of antibiotics, which was previously shown to alter the composition of gut microbiota in mice [40], can increase CRC risks [41,42]. 

Overall, the findings described above suggest that CRC development appears to be linked to changes in the levels of various bacterial species in the intestine. The next question is how compositional changes in intestinal microbiota may contribute to CRC progression.

### 2.2. Current Evidence on the Mechanisms of How Microbial Dysbiosis Can Induce CRC 

#### 2.2.1. Inflammation

Chronic inflammation has long been considered to play a contributory role in the promotion of carcinogenesis [43,44]. Indeed, inflammatory bowel disease, a condition that involves the inflammation of the gut, has been described as a risk factor in the development of CRC [45]—that gut inflammation can increase CRC risks. Further, it has been shown that fewer tumours would develop in a mouse model of CRC (APC^min^ mice) that was deficient in toll-like receptor signalling, a signalling pathway that plays a role in the initiation of inflammation [46]. This finding suggests that inflammation is at least partly involved in tumour development. The finding that regular and long-term intake of aspirin, a non-steroidal anti-inflammatory drug (NSAID) that relieves inflammation, reduces CRC risk further supports the contributory effect of chronic inflammation to CRC [47,48]. During the inflammatory process, immune cells such as macrophages step up the production of reactive oxygen species (ROS) and reactive nitrogen species (RNS) [49], which may cause damage to DNA and the mutation of genes involved in cell cycle control and DNA repair. Further, proinflammatory cytokines produced by activated macrophages, such as interleukin-1β (IL-1β), have been shown to activate the Wnt signalling pathway, which is a prerequisite for β-catenin-mediated gene expression promoting the survival of colon cancer cells [50]. Taken together, these data suggest that chronic inflammation creates an optimal environment for tumour initiation and growth. 

In fact, there is evidence to support the role of microbial dysbiosis in the promotion of inflammation, as certain pathogenic bacterial species have been shown to contribute directly to inflammatory events. A bacterial species belonging to the *Enterococcaceae* family (*Enterococcus faecalis*) was found to be able to polarise colon macrophages to the M1 phenotype, causing them to increase the expression and release of proinflammatory cytokines such as tumour necrosis factor-α (TNF-α). This would lead to the activation of the Wnt signalling pathway and the promotion of carcinogenesis [51]. In addition to *Enterococcaceae*, *Fusobacteria* were also described as promoters of inflammation. Oral administration of *Fusobacteria* in APC^min^ mice was shown to result in an increase in myeloid cell infiltration into the tumours in these mice, coupled with an increase in the expression of proinflammatory cytokines [52]. The promotion of inflammation by *Fusobacteria* is likely to be associated with the pathogenesis of CRC, as evidenced by the finding that the abundance of *Fusobacteria* in CRC patients correlates with the expression of genes coding for TNF-α [53]. Moreover, as discussed above, *Fusobacteria* may induce the increased expression of FadA in colon tissues, which has been demonstrated to lead to an increase in the production of proinflammatory cytokines, contributing to intestinal chronic inflammation [27]. Likewise, the *Streptococcus gallolyticus*-enriched colon tissues taken from CRC patients have been shown to exhibit an increased expression of genes involved in inflammation, including interleukin-1, interleukin 8, and cyclooxygenase 2 [31]. Further, in CRC patients, changes in the composition of intestinal microbiota are accompanied by an increase in the number of cells that produce interleukin-17 [29], a proinflammatory cytokine implicated in chronic inflammatory diseases [54], in the intestinal mucosa. Consistent with these findings, the blocking of interleukin-17 by antibodies was found to relieve colitis induced by enterotoxigenic *Bacteroides fragilis*, and inhibit the formation of tumours, in a CRC mouse model [55]. Moreover, the removal of *Helicobacter typhlonius*, a bacterial species found to cause intestinal inflammation in immunodeficient mice [56], from mouse intestines using antibiotics was demonstrated to result in a reduced number of intestinal tumours in these mice [57]. All these findings add weight to the hypothesis that the modification of intestinal microbiota is associated with CRC via proinflammatory conditions. 

In contrast, one recent study suggests that health-promoting bacteria may contribute to the lessening of tumourigenesis by reducing inflammation. The study revealed that the administration of probiotic bacteria (*Clostridium butyricum* and *Bacillus subtilis*) to mice treated with the carcinogenic 1,2-dimethylhydrazine dihydrochloride inhibited CRC development in them, an effect partly attributed to the reduction of intestinal inflammation [58]. Nevertheless, further research is required to provide more substantial evidence of the role of health-promoting bacteria in the inhibition of tumourigenesis via the reduction of inflammation. 

Taken together, previous studies suggest that chronic intestinal inflammation plays a role in the pathogenesis of CRC, and the balance in the levels of pathogenic and health-promoting bacteria in the gut may modify CRC risks by moderating the extent of intestinal inflammation.

#### 2.2.2. Induction of DNA Damage

In addition to the promotion of inflammation, pathogenic bacteria were also reported to employ mechanisms leading to DNA damage in order to promote carcinogenesis. *Enterococcus* *faecalis*, a bacterial species found to be enriched in stool samples of CRC patients [30], was previously shown to possess the ability to generate superoxide radicals [59]. The increased production of these free radicals was shown to cause DNA damage in colonic epithelial cells, thereby leading to gene mutations and CRC induction [60]. More recently, pathogenic strains of *Escherichia coli* that harbour a genomic island called pks were found to be able to produce such genotoxins as colibactin. Indeed, colibactin was shown to induce DNA damage, leading to mutations and chromosomal instability. In vitro infection with colibactin-producing *Escherichia coli* was also shown to result in cells exhibiting a higher degree of DNA damage and more frequent gene mutations [61]. This suggests that there is an increased risk of CRC development upon the increased colonisation of such *Escherichia coli* strains in the colon. This hypothesis is further supported by the recent finding that *Escherichia coli* strains possessing the pks islands were over-represented in the biopsy colon mucosal samples of CRC patients compared with those of controls [62]. Therefore, changes in the intestinal microbiota could also contribute to CRC development through DNA damage in colonocytes induced by free radicals and genotoxins.

In fact, there appears to be a relationship between the chronic inflammatory process and DNA damage caused by microbial dysbiosis. During inflammation, immune cells such as macrophages will step up the production of free radicals like ROS or RNS [63], in an attempt to remove pathogens or tumours by a process known as respiratory burst. Meanwhile, these cells will increase the production of proinflammatory cytokines, which may contribute to DNA damage through oxidation [64]. Chronic inflammation may therefore lead to oxidative stress and the accumulation of free radicals [65], a process known to be linked to carcinogenesis [66]. 

To summarise, previous findings suggest that microbial dysbiosis may contribute to alteration in the composition of the intestinal microbiota. Such alteration is likely to contribute to chronic inflammatory conditions in the gut, and the increased production of genotoxins and free radicals from resident immune cells, which causes an increased level of oxidative stress and increases the likelihood of DNA damage. It is considered that such conditions will provide an optimal environment for tumour growth in the intestine, and lead to an increased risk of CRC development.

### 2.3. Dietary Rice Bran Intake, Promotion of a Healthy Intestinal Microbiota, and Protection against CRC

As discussed, one of the possible mechanisms of CRC pathogenesis consists of changes in the composition of intestinal microbiota, favouring the colonisation of pathogenic bacteria that outnumber health-promoting species, and resulting in intestinal chronic inflammation and local DNA damage. Measures that can modify the intestinal microbiota in a way that ensures the presence of a significant proportion of health-promoting bacteria in the gut may therefore be potentially effective anti-CRC strategies. With currently emerging studies demonstrating health-promoting changes in the composition of the intestinal microbiota after rice bran intake in both humans and animals, it is tempting to speculate that rice bran consumption may lead to CRC chemoprevention through modification of the intestinal microbiota. The relationship between dietary rice bran intake, composition of intestinal microbiota, and CRC prevention is therefore an active area of current investigation. We provide here an overview of studies showing the effect of rice bran intake on intestinal microbiota, and of current evidence demonstrating the relationship set out above.

Previous research efforts have investigated how rice bran intake could modify intestinal microbiota, and whether or not it would confer some form of protection against CRC. In this review, primary studies on this topic were identified through a literature search on PubMed, using keywords that included “rice bran” and “microbiota”. A summary of these studies is presented below. Earlier studies on the topic largely used mice to study the effect of dietary supplementation by rice bran on the composition of gut microbiota. For example, diet supplementation with rice bran in mice resulted in an increase in the intestinal colonisation of *Lactobacillus* [67,68], a bacterial genus shown to protect against CRC [69]. To lend support to this observation, mice fed a diet supplemented by rice bran oil were also found to contain a significantly higher abundance of *Lactobacillus* in their intestines compared to the controls [70]. Furthermore, Henderson and colleagues found that the increase in *Lactobacillus* colonisation in mouse intestines from rice bran intake was accompanied by increased production of immunoglobulin A (IgA) [67], indicating the effect of dietary rice bran on enhancing intestinal mucosal immunity, which had previously been thought to be involved in the prevention of CRC progression [71].

In addition to mice, the effect of rice bran intake on the composition of intestinal microbiota was also demonstrated in other animal species. For example, a recent study showed that rice bran intake would lead to a significant expansion of the probiotic species, such as *Lactobacillus rhamnosus GG* and *Escherichia coli Nissle*, in the intestines of pigs [72]. In contrast, in a study using a colitis model in rats, the investigators showed that enzyme-treated rice fibre, a product derived from rice bran through enzymatic treatment, would significantly suppress the growth of a pathogenic *Clostridium* species. This was also accompanied by an increase in the production of butyrate in the colon of the rats, followed by amelioration of colonic inflammation in the rats [73]. In this way, the findings provide evidence that rice bran intake may contribute to a reduction in colon inflammation, an event associated with CRC.

More recently, a dietary intervention for human subjects was carried out with heat-stabilised rice bran, and revealed that the modification of intestinal microbiota resulting from rice bran intake also applied to humans. In an American study, subjects who consumed rice bran for a period of four weeks were found to have a significantly higher level of *Bifidobacterium* and *Ruminococcus*, the two health-promoting bacteria genera, in their stool samples [74]. This observation was also accompanied by the presence of beneficial chemicals in the samples, including cycloartenol and β-sitosterol, which were shown to have an inhibitory effect on carcinogenesis in mice and antioxidative properties, respectively [75,76]. In addition, increase in the production of branched chain fatty acids and bile acids was observed in the subjects who completed the rice bran dietary intervention [74]. In another study, intake of rice bran for 14 days was shown to result in a decreased ratio of the intestinal abundance of *Firmicutes* and *Bacteroidetes* (decreased Firmicutes–Bacteroidetes ratio), as well as an increased production of some health-promoting SCFAs such as acetate, among American CRC survivors [77]. This is consistent with the recent finding that the intake of feruloylated arabinoxylan oligosaccharides, a carbohydrate contained in rice bran [78], would lead to an elevation in the intestinal production of SCFAs including butyrate and propionate [79]. These SCFAs were previously suggested to exhibit cancer-preventive properties [17]. These findings therefore suggest a potential mechanism by which rice bran consumption can confer intestinal health and CRC prevention. Interestingly, the consumption of whole grain brown rice, which contains the bran layer where rice bran is derived from, was found to lead to an increase in Firmicutes–Bacteroidetes ratio in healthy subjects. This effect was accompanied by an increase in the faecal abundance of the health-promoting *Blautia* genus and a modest reduction of the plasma level of proinflammatory cytokine interleukin-6 [80]. The discrepancy in the findings by Sheflin et al. [77] and Martinez et al. [80] is possibly due to differences in the nature of the subjects and the dietary supplement utilised in their studies. Therefore, data from animal and human studies suggest that rice bran intake can lead to changes in the microbial composition in the intestine. In addition, such intake not only can suppress intestinal inflammation by favouring the intestinal colonisation of health-promoting bacteria, but also inhibit oxidative stress. A summary of the studies that provide evidence of the link between rice bran intake and modification of intestinal microbiota is presented in Table 1.

In fact, the chemopreventive properties of rice bran are not limited to altering the composition of intestinal microbiota, but also lead to a reduction in the metabolic activity of intestinal bacteria detrimental to colon health. Certain bacterial metabolic activities have been shown to contribute to colon carcinogenesis. One example is β-glucuronidase activity, demonstrated by the finding that the reduction of this activity by administering *Bifidobacterium* to rats appears to lower the number of colonic aberrant crypt foci in these animals [81]. This finding has also provided an insight into the possible mechanism that *Bifidobacteria* employ in exhibiting health-promoting effects in the intestines. Indeed, the administration of the carcinogenic 1,2-dimethylhydrazine in rats was found to increase the activity of colonic bacterial enzymes, including β-glucuronidase. However, the supplementation of p-methoxycinnamic acid, a phytochemical present in rice bran, in the rats’ diet would reverse the effect of 1,2-dimethylhydrazine on β-glucuronidase activity [82], suggesting that rice bran intake can also confer beneficial effects by regulating the β-glucuronidase activity of intestinal bacteria. 

**Figure 1:**
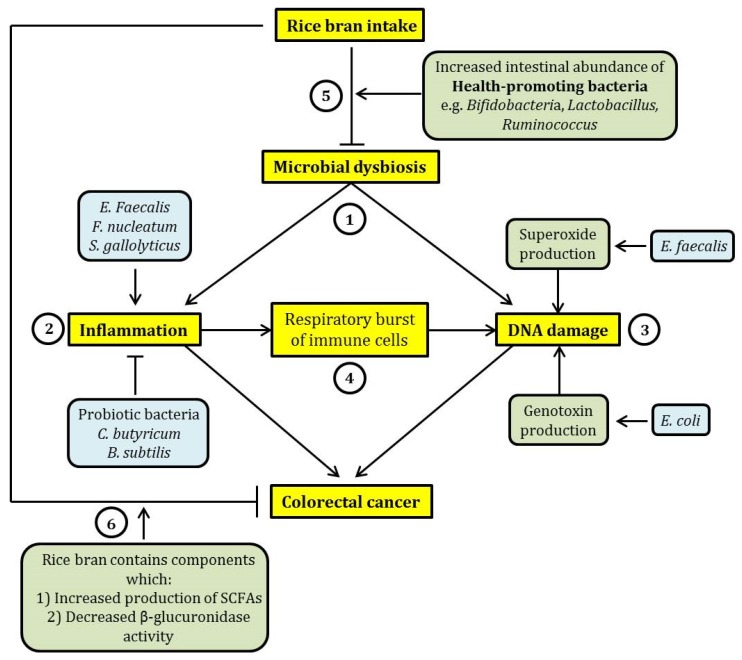
A schematic summary of the relationship between dietary rice bran intake, microbial dysbiosis and colorectal cancer (CRC). (**1**) Microbial dysbiosis may lead to CRC mainly through the induction of intestinal inflammation and DNA damage. (**2**) Inflammation may be exacerbated by certain pathogenic intestinal bacteria such as *Enterococcus* *faecalis*, *Fusobacteria nucleatum,* and *Streptococcus gallolyticus*, while probiotic bacteria (*Clostridium butyricum* and *Bacillus subtilis*) exhibit an opposing effect. (**3**) DNA damage induced by intestinal bacteria can be elicited through the increased production of free radicals such as superoxide (by *Enterococcus* *faecalis*) and production of genotoxins such as colibactin (by *Escherichia coli*). (**4**) Further, inflammation may lead to an increase in proinflammatory cytokine production by immune cells through respiratory burst. This effect may cause DNA damage through oxidation. This suggests a relationship between inflammation and DNA damage induced by microbial dysbiosis. (**5**) The consumption of rice bran may reverse microbial dysbiosis through the expansion of the population of health-promoting bacteria, such as *Bifidobacteria* and *Lactobacillus*, in the intestine. This may potentially ameliorate the detrimental and cancer-causing effects of microbial dysbiosis. (**6**) In addition, rice bran intake has a beneficial effect on intestinal health because rice bran contains chemicals that were previously shown to exhibit cancer chemo-preventive effect, by eliciting an increased production of short-chain fatty acids (SCFAs) and inhibiting the activity of β-glucuronidase. In the figure, arrow-headed lines indicate “promotion” and bar-headed lines indicate “inhibition”.

As an overall summary, microbial dysbiosis in the intestine would mainly lead to CRC via intestinal inflammation and the induction of DNA damage. Intestinal inflammation can be contributed by the increased intestinal abundance of pathogenic bacterial species. However, the increased presence of health-promoting probiotic bacteria may ameliorate intestinal inflammation. This therefore indicates the importance of the expansion of the intestinal population of probiotic bacteria in the reduction of intestinal inflammation. Pathogenic bacteria such as *Enterococcus faecalis* and *Escherichia coli* may also contribute to DNA damage via the increased production of superoxide free radicals and genotoxins, respectively. In addition, intestinal inflammation may further contribute to DNA damage through the induction of respiratory burst in immune cells, promoting the release of ROS and RNS which can cause DNA damage. The intake of rice bran would potentially ameliorate these molecular events as it may lead to an increased intestinal abundance of health-promoting bacteria, counteracting microbial dysbiosis. In addition, rice bran intake may lead to an increased intestinal production of SCFAs and the reduction of cancer-causing enzymatic activities of certain intestinal bacteria. In other words, rice bran would exert beneficial effects on intestinal health and potentially CRC prevention via an interaction with the intestinal microbiota. A graphical representation of the relationship between dietary rice bran intake, the compositional changes of intestinal microbiota and CRC is shown in Figure 1.

## 3. Research into Link between Rice Bran and CRC Prevention: What Next? 

Despite the fact that past studies have suggested the role of rice bran intake in modifying the composition of intestinal microbiota, and thus modifying CRC risks, the body of evidence is currently not large and not conclusive enough to establish whether rice bran intake really can achieve CRC chemoprevention through encouraging a healthy composition of intestinal microbiota. To address this, further studies are required to investigate whether rice bran intake would result in a reduction in the intestinal level of certain bacterial species previously shown to be associated with CRC risks. To date, most studies on the effect of rice bran intake on the composition of intestinal microbiota have pointed towards expansion of health-promoting bacteria, such as *Lactobacillus* and *Bifidobacteria*, in the intestine. Few studies have indicated a decreasing level of CRC-associated bacterial genera or species as a result of rice bran intake (Table 1). As indicated above, a number of pathogenic bacterial species or genera have been revealed to exhibit increased colonisation in the colon of CRC patients and animal models, and thought to play a role in CRC carcinogenesis. These include *Fusobacteria* *nucleatum*, *Bacteroides fragilis*, *Enterococcus faecalis*, and *Streptococcus gallolyticus*. Moreover, to the best of our knowledge, no studies have previously been carried out to investigate changes in the intestinal level of bacteria known to be producers of butyrate, the SCFA that leads to intestinal health, such as the bacterial species belonging to the Clostridial clusters IV and XIVa [18]. Therefore, to come to a better understanding of whether rice bran intake can reduce CRC risks, analysis is required of changes in the levels of these specific intestinal bacterial species/genera after rice bran intake. In the light of this, rice bran dietary interventions should be carried out among cancer patients, to assess whether consumption would lead to a change in the intestinal abundance of the above pathogenic and butyrate-producing bacteria in these patients to a level comparable to that of healthy controls. This would provide further evidence of whether an inter-relationship exists between rice bran intake, the composition of intestinal microbiota and CRC.

Furthermore, with only few recent studies reporting changes in the levels of certain bacterial genera in the human intestine after a rice bran dietary intervention (Table 1), research into how rice bran intake can modify the intestinal microbiota of humans is currently at a very early stage. More data describing such effects on humans of varying ethnicity are recommended, to obtain a more comprehensive picture of the effect of rice bran intake on the composition of human intestinal microbiota.

## 4. Concluding Remarks

Although there are a great many studies showing a close relationship between the composition of intestinal microbiota and CRC development, the beneficial effect of rice bran intake on such modification, and how this contributes to chemoprevention in the case of CRC development and progression, is still currently an area that requires further investigation. Nevertheless, evidence is currently emerging that rice bran intake would help to modify the composition of intestinal microbiota, through the amplification of the population of health-promoting bacteria demonstrated in both humans and animal models. Emerging evidence also points towards the health-promoting effect of rice bran consumption through the increased production of SCFAs. Currently, data are still scarce on the beneficial effects of rice bran intake among humans, both by inhibiting tumourigenesis and by modifying intestinal microbiota. However, recent findings on the effect of rice bran intake on human intestinal microbiota highlight an encouraging prospect of its use in preventing CRC. 

However, it is important to note that increased rice bran consumption may also bring about certain negative effects on health. For example, as revealed by Sheflin et al. [74], the intake of rice bran would lead to a slightly increased (1%–3%) production of certain bile acids, such as deoxycholic acid and lithocholic acid. These bile acids were previously demonstrated to exhibit a cancer-promoting effect owing to their contribution to the development of oxidative stress [83]. Further, rice bran was shown to contain trace amounts of inorganic arsenic, which is a carcinogen, possibly through environmental pollution of water used for growing rice [84]. On balance, however, the well-established chemopreventive effect of rice bran on CRC should outweigh these negative effects as long as the daily dosage of intake is optimal.

In the light of the benefits of rice bran intake in CRC prevention, more research efforts should be directed towards uncovering further evidence for the hypothesis that rice bran intake brings CRC chemoprevention through modulation of intestinal microbiota. Dietary intervention studies should also be carried out to investigate these effects among individuals of various ethnic backgrounds to confirm the effects of rice bran intake on the composition of intestinal microbiota. Findings from such studies would ultimately establish the applicability of rice bran dietary interventions to CRC prevention, and provide a scientific basis for the development and refining of a dietary intervention in an attempt to achieve more effective CRC prevention for the global population.

## Figures and Tables

**Table 1:** A summary of evidence that rice bran intake can lead to a change in the composition of intestinal microbiota.

Subject of Study	Bacterial Phyla/Genera/Species Involved	Nature of Bacteria	Main Findings	References
Mouse	*Lactobacillus*	Health-promoting	Mice exhibited a 500% increase in lactobacilli colonisation after 11 days of rice bran intake.	Henderson et al., 2012 [67]
Mouse	*Lactobacillus*	Health-promoting	Mice fed with rice bran exhibit a 170-fold increase in faecal *Lactobacilli*.	Kumar et al., 2012 [68]
Mouse	*Lactobacillus*	Health-promoting	A significant increase in the occupational ratio of lactobacillales in mice fed with rice bran oil.	Tamura et al., 2012 [70]
Pig	*Lactobacillus rhamnosus GG Escherichia coli Nissle*	Health-promoting	10^4^–10^5^ increase in the numbers of the studied bacterial species from the intestine of pigs fed with rice bran for 30 days.	Yang et al., 2015 [72]
Rat	*Clostridium*	Pathogenic	Intake of enzyme-treated rice fibre, a product derived from rice bran through enzymatic treatment, significantly suppressed the growth of *Clostridium* in rat intestines.	Komiyama et al., 2011 [73]
Human	*Bifidobacterium sp* *Ruminococcus bromii Ruminococcus flavefaciens*	Health-promoting	Significant increases in levels of the studied bacterial species from the stool samples of human subjects taking rice bran for 2 or 4 weeks.	Sheflin et al., 2015 [74]
Human	*Firmicutes Bacteroidetes*	Not applicable	Decreased *Firmicutes*-*Bacteroidetes* ratio in the intestine of CRC survivors upon the intake of rice bran for 14 days, at a dose of 30 g per day.	Sheflin et al., 2016 [77]
Human	*Blautia*	Health-promoting	An increase in faecal abundance of *Blautia* upon the intake of brown rice for four weeks among human subjects, at a dose of 60 g per day. An increase in their *Firmicutes*-*Bacteroidetes* ratio was also observed as a result of the intervention.	Martínez et al., 2013 [80]
